# Microvascular complications and its predictors among type 2 diabetes mellitus patients at Dessie town hospitals, Ethiopia

**DOI:** 10.1186/s13098-021-00704-w

**Published:** 2021-08-17

**Authors:** Mohammed Abdu Seid, Yonas Akalu, Yibeltal Yismaw Gela, Yitayeh Belsti, Mengistie Diress, Sofonias Addis Fekadu, Baye Dagnew, Mihret Getnet

**Affiliations:** 1 Unit of Human Physiology, Department of Biomedical Science, College of Health Sciences, Debre Tabor University, P. O. Box: 272, Debre Tabor, Ethiopia; 2 Department of Human Physiology, School of Medicine, College of Medicine and Health Sciences, University of Gondar, Gondar, Ethiopia; 3 Department of Optometry, College of Medicine and Health Sciences, University of Gondar, Gondar, Ethiopia

**Keywords:** Microvascular complications, Predictors, Type 2 diabetes mellitus, Ethiopia

## Abstract

**Background:**

Diabetes mellitus is a serious metabolic disorder which becomes common in middle and low incomes countries since few decades. Microvascular complications include retinopathy, neuropathy and nephropathy all of which can lead to disability, dependency, accelerate their morbidity, and mortality. In Ethiopia, there is paucity data regarding this topic. Hence, this study aimed to assess prevalence of microvascular complications and its predictors among type 2 diabetes mellitus patients.

**Methods:**

Cross-sectional study was conducted from February to March 2020 at Dessie town hospitals. We used simple random sampling to recruit study participants and pre-tested interviewer administered questionnaire to collect the data. Data was entered into Epi-Data 3.1 and exported to SPSS-23 for analysis. Binary logistic regression was done to select potential variables to be adjusted at p ≤ 0.25. After running multivariable regression, variables with a p-value ≤ 0.05 were declared as statistically significant.

**Results:**

Three hundred and thirty-five type 2 DM patients participated in the study, of which 54.6% were males. One hundred and twenty-seven [37.9% (95% CI 32.5%–43.3%)] of diabetes mellitus had at least one microvascular complications. These were retinopathy 24.8%, nephropathy 16.1%, and neuropathy 8.1%. Age 60–87 years (AOR = 2.76, 95% CI 1.02–7.46), duration of diabetes > 5 years (AOR = 4.09, 95% CI 2.40–6.96), mellitus and co-morbid hypertension (AOR = 3.52, 95% CI 2.09–5.95), were statistically significant.

**Conclusions:**

In this study, diabetic microvascular complications are prevalent. Increasing the age of participants, longer duration of diabetes mellitus and co-morbid hypertension were independent predictors. Health workers should give emphasis for diabetes mellitus through early screening and health education, abrupt medication for aged patients with long duration of diabetes mellitus, and hypertension, and also early detection and management of microvascular complication.

## Background

Microvascular complications are sequels of diabetes mellitus following uncontrolled chronic hyperglycemia which includes diabetic nephropathy, neuropathy and retinopathy, that are caused by pathological changes in capillaries [1, 2]. The time to develop microvascular complications is much faster and common than macrovascular complication [3].

Diabetes mellitus (DM), commonest metabolic illness, is one of the major public health concern worldwide [4]. Diabetes burden has been rising more rapidly in low and middle income countries than in high income countries [3, 5], which is attributed to the effect of globalization, life style modification (change in diet type and pattern) and physical inactivity (being obese) [6].

Nowadays, the number of peoples living with diabetes increases rapidly and the disease become pandemic. Since few decades, the prevalence of type 2 diabetes has increase radically in all countries of the globe. It is about 422 million people have diabetes mellitus worldwide, and low-and middle-income countries are majorly the victims [5]. The disorder is also predicted to 578 million in 2030 and inflated to 700 million by the year 2045 [7]. In other words, nearly 1 in every 11 adults has diabetes globally, of which more than 3/4th is type 2 DM [3]. Morbidity and mortality in chronic diabetes mellitus is now common that is more than four million people aged 20–79 years were estimated to die from diabetes related complications [7]. Adequate data on tendencies of microvascular diabetes complications and other evolving complications are lacking and so that conclusions are incomplete [8].

Following the increase number of type 2 DM, its microvascular complication is rising proportionally and substantially [9]. Diabetic microvasculature is highly susceptible for damage due to chronic hyperglycemia and genetic predisposition, leading to complications of essential organs such as the kidneys, the eyes and the nervous system. Diabetic nephropathy is the foremost cause of serious renal disease, diabetic retinopathy (DR) is the amongst cause of blindness in diabetes population and that of diabetic neuropathy is the main attributing factor for diabetic foot ulcer and amputation [9, 10].

Almost all organs are affected and people suffer from serious morbidity and mortality due to type 2 DM related complications. The commonest type 2 DM microvascular complications are retinopathy, neuropathy and nephropathy that are present at the time of patient diagnosis [4, 11]. Chronic diabetes prone to lose more than half of the direct health costs following complications [12]. Indeed, they are eligible to develop disability, accelerate mortality and fail to avail work on regular basis due to the illness [1, 8, 13].

Globally, around 1/5th (18.8%) of type 2 diabetes mellitus developed microvascular complications [14], and this proportion was increased to 45% in middle east [15] and 47.8% in African diabetes [16]. In particular, different studies were conducted in different countries and revealed varying prevalence which is supported in USA 77% [17], Brazil 41.6% [18], China 57.5% [19], Spain 25.2% [20], Bangladesh 50.4% [21], Greenland 68% [12], Kuwait 61.6% [22], Saudi Arabia 35.4% [23], and Ghana 35.3% [24].

The emerging prevalence of diabetes and its complication also noted in Ethiopia. For instance, microvascular complication of type 2 diabetes is prevalent in Gondar, Ethiopia 20.4% [25], wollega hospitals 31.2% [26], Gurage zone 61% [27], Jimma university hospital 41.5% [28], Mettu Karl Referral Hospital 38.5% [29], and Debre tabor Hospital 43.9% [30].

Different literature showed that a number of factors are associated with microvascular diabetic complications. These factors could be grouped as socio-demographic factors (age, sex, and marital status), behavioral factors (obesity, diet) and clinical factors (glycemic control, and duration of diabetes, comorbidities (hypertension) and medication). In particular studies, being female [28, 31], age [14, 16, 19, 22, 26, 30, 32, 33], marital status (single or divorced) [19, 27], family history of diabetes mellitus [15, 26, 28, 31], longer duration of diabetic [9, 16, 19, 22, 23, 26, 27, 30–32, 34], hypertension [9, 16, 18–20, 22, 23, 26, 27, 30, 32, 34, 35], obesity [22, 27, 31], poor glycemic control [18, 22, 25, 27, 28, 33, 34], adherence to diet [16, 22, 35], mixed medication [26, 30] and insulin therapy only [22] were predictors for microvascular complications among type 2 diabetes mellitus patients.

In Ethiopia, people living with type 2 DM ranged 2% to 7% and its long term sequels are the major causes of morbidity and mortality besides to its economic crisis and social stigma [36]. Literature sources that showed factors exacerbating diabetic complications were too limited. Hence, this study was aimed to assess prevalence of microvascular complications and its predictors among type 2 diabetes mellitus patients in Dessie town hospitals.

## Methods and materials

### Study design, setting and period

Cross-sectional study was conducted from February to March 2020 at Dessie town hospitals. Dessie Town is found 400 km Northeast of Addis Ababa, the capital town of Ethiopia. Around fourteen thousand diabetes patients were served in a total of 5 hospitals. Diabetes patients have follow-up dates from Monday to Friday.

### Study population and eligibility

Type 2 diabetes clients visiting the Hospitals at a regular base (every 3 months) as outpatient and available during the period of data collection were participants and eligible for this study. Those individuals living with diabetes who were critically ill or unconscious, gravid women at 2nd trimester and above were excluded.

### Determination of sample size and sampling

Sample size was estimated considering the following assumptions: p = 0.705 [33], 95% CI, and 5% margin of error. Hence, the calculated sample size was 319 and by adding 5% oversampling to account for non-respondents, we got a total of 335. We used simple random sampling technique to recruit study participants and allocated proportionally for each hospital of Dessie town.

### Study variables

- Dependent variable: Microvascular complications
- Independent variable: Socio-demographic factors (age, sex, residence, marital status, occupation), behavioral factors (physical activity, obesity, diet) and clinical factors (glycemic control, and duration of diabetes mellitus, comorbidities (hypertension) and anti-diabetic medication)

### Operational definitions

Microvascular complications: Diabetes mellitus patients with one or more of the following complications: diabetic nephropathy, diabetic retinopathy, and peripheral neuropathy [14] of known diabetes or newly diagnosed diabetes.

Physical activity: Diabetes mellitus individuals who perform at least 150 min per week (3 days) of moderate intensity exercise regarded as *Good* otherwise *poor* physical activity [37].

Glycemic control level: It was *good* glycemic control when fast blood sugar (FBS) was below 130 mg/dL and above indicated value was regarded as *poor* [38].

Obesity: A client with body mass index (BMI) of ≥ 30 kg/m^2^. It has three categories, category 1 (BMI: 30–34.9 kg/m^2^), category 2 (BMI:35–39.9 kg/m^2^) and category 3 or extreme obesity ( BMI: ≥ 40 kg/m^2^) [39].

Adherence to diet: Diabetes mellitus individuals adjust life style (diet) as recommended for more than 3 days in last seven consecutive days.

### Data collection instrument, procedure and quality control

Data were collected using a semi-structured interviewer administered questionnaire consisting socio-demographic factors, behavioral & clinical predictors of microvascular complication in type 2 diabetes. These complications were diagnosed based on the physical, clinical, laboratorial and other requested findings and decision by the physician. Fundus ophthalmoscopy examination (the presence of neovascularization, hemorrhage spot, vitreous hemorrhage, microaneurysm, macular lesion and cotton wool spot) was done to diagnose diabetic retinopathy. For neuropathy, clinical assessment like history of numbness, paresthesia, tingling sensation and tests for vibration sensation was used. Likewise, symptoms such as swelling of feet, hands or eyes, urinary frequency and urgency, BP measurement and tests like protein in the urine, renal function tests and ultrasound were used diabetic nephropathy. Participants were interviewed and their medical chart was reviewed by trained data collectors to determine clinical and related factors. Clients’ weight, height and blood pressure was measured in line with standard protocol. Data quality was assured by giving training prior to data collection and data collectors were supervised and applied measurements at least twice and the average was taken with the nearest value of 0.01. Routinely, diabetes patients who had follow-up in diabetic clinic of each hospital were ordered to come to the next visit with their FBS result and data collectors used the newly FBS result. The data collection tools and instruments used were developed by for this study after reviewing different related literatures.

### Reliability and validity

The Cronbach’s alphas for this study was below 0.5 which is due to the questionnaires we used were self-developed and unstandardized. Content validity was ensured by pre-testing the data collection tool on 5% of diabetes. The tool was modified based on the observed findings from the pre-test result. Some questions having ambiguous meaning were rewritten for better understanding of study participants.

### Data processing and statistical analysis

Data were cleaned, coded and entered into Epi-Data 3.1 and exported into SPSS version 23 for statistical analysis. Frequency tables with percentage, median, and interquartile range (IQR) was used to describe study findings. Variables with p-value ≤ 0.25 in the binary logistic regression (age, marital status, residence, educational level, occupation, duration of diabetes, medication regimen, physical activity, hypertension and adherence to diet) were entered into multivariable logistic regression analysis. We used crude odds ratio (COR) and adjusted odds ratio (AOR) at 95% to determine the strength of association. Model fitness was verified by Hosmer and Lemeshow model fit and variance inflation factor tested variables multicollinearity. Lastly, variables with a p-value ≤ 0.05 were declared as statistically significant.

## Results

### Socio-demographic characteristics of participants

A total of 335 type 2 diabetes mellitus were took part in the study. The median age of participants was 53 years (IQR: 45–60 years). More than half of the clients [183 (54.6%)] were males. Majority of individuals [302 (90.1%)] were married. Quarter of [78 (23.3%)] them did not attend formal education and three-forth [259 (77.3%)] individuals were urban dwellers (Table 1).

**Table 1:** Socio-demographic characteristics of type-2 diabetes mellitus at Dessie town hospitals, Ethiopia 2020 (n = 335)

Variable	Categories	Frequency (%)	Microvascular complications	
			No	Yes
Age	20–39 years	37 (11.0)	30	7
	40–59 years	195 (58.2)	139	56
	60–87 years	103 (30.8)	39	64
Sex	Male	183 (54.6)	118	65
	Female	152 (45.4)	90	62
Marital status	Never married	33 (9.9)	24	9
	Married	302 (90.1)	184	118
Residence	Urban	259 (77.3)	174	85
	Rural	76 (22.7)	34	42
Education	No read and write	78 (23.3)	31	47
	Grade 1–8	84 (25.1)	53	31
	Grade 9–10	82 (24.5)	59	23
	Certificate and above	91 (27.2)	65	26
Occupation	Employed	73 (21.8)	49	24
	Private workers*	117 (34.9)	83	34
	Peasants	37 (11.0)	18	19
	House wife	65 (19.4)	34	31
	Others**	43 (12.9)	24	19

Private worker*: manufacturer, daily laborer, vehicle driver, mechanic, and wholesalers

Others**: unemployed and retiree

### Prevalence of microvascular diabetic complications

**Fig. 1:**
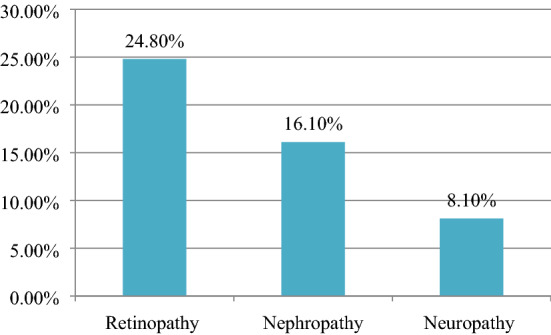
Proportion of microvascular diabetic complications among type-2 diabetes mellitus at Dessie town hospitals, Ethiopia 2020 (n = 335)

In this study, the prevalence of at least one microvascular diabetic complications was 37.9% (95% CI 32.5%-43.3%). Specifically, the prevalence of retinopathy, 24.8% (95% CI 20.3–29.6%), nephropathy, 16.1% (95% CI 12.2–20.3%), and neuropathy, 8.1% (95% CI 5.1–11%) among type 2 diabetes mellitus (Fig. 1).

### Behavioral and clinical characteristics of type 2 diabetes mellitus patients

In this study, majority of individuals [269 (80.3%)] and [205 (61.2%)] had no family history of diabetes and diabetic education of ≤ 5 years respectively. The drugs of choice for 223 (66.6%) type 2 DM patients were oral drugs. Two hundred and eight (62.1%) diabetes had inadequate physical activity. Majority of them, [297 (88.7%)] and [287 (85.7%)] were non-obese and had poor glycemic control respectively. Only quarter [66 (19.7%)] of individuals has adherence to diet and 231 (69.0%) diabetes had co-morbid hypertension (Table 2).

**Table 2:** Behavioral and clinical characteristics of type-2 diabetes mellitus patients at Dessie town hospitals, Ethiopia 2020 (n = 335)

Parameters	Categories	Number	Percentage
Family history of diabetes	Yes	66	19.7
	No	269	80.3
Duration of diabetes	≤ 5 years	205	61.2
	> 5 years	130	26.4
Medication regimen	Drugs (OHA)	223	66.6
	Both oral & insulin	84	25.1
	Insulin only	28	8.3
Physical activity	Good*	127	37.9
	Poor**	208	62.1
Obesity	Yes	38	11.3
	No	297	88.7
Hypertension	Yes	104	31.0
	No	231	69.0
Adherence to diet	Yes	66	19.7
	No	109	84.5
Glycemic control	Good	48	14.3
	Poor	287	85.7

*OHA* oral hypoglycemic agent, Good* perform at least 150 min/week (3 days) of moderate intensity exercise, Poor** had not exercised at all or perform less than 150 min/week

### Predictors of microvascular complications among type 2 diabetes mellitus patients

In bi-variable logistic analysis, age, marital status, residence, educational level, occupation, duration of diabetes, medication regimen, physical activity, co-morbid hypertension and adherence to diet had p-value ≤ 0.2 and hence were entered in to multivariable binary logistic analysis. In the final model, age of participants, duration of diabetes and co-morbid hypertension were statistically significant with the occurrence of microvascular complications.

The odds of developing at least one diabetes microvascular complications for age groups 60–87 yeas was 2.76 times (AOR = 2.76; 95% CI 1.02–7.46) higher as compared to younger diabetes aged 20–39 years. Participants who had a duration of diabetes more than 5 years was 4 times (AOR = 4.09; 95% CI 2.40–6.96) more likely to have microvascular complications in contrary to duration of diabetes 5 years or less. Moreover, diabetic clients with co-morbid hypertension were 3.52 times (AOR = 3.52; 95% CI 2.09–5.95) more likely to acquire microvascular complications than non-hypertensive diabetes (Table 3).

**Table 3:** Multivariable binary logistic analysis for factors associated to microvascular complications among type-2 diabetes mellitus at Dessie town hospitals, Ethiopia 2020 (n = 335)

Parameters	Categories	Microvascular complications		OR	
		No	Yes	COR (99% CI)	AOR (95%CI)
Age	20–39 years	30	7	1	1
	40–59 years	139	56	1.72 (0.71–4.16)	1.18 (0.46–3.01)
	60–87 years	39	64	7.03 (2.82–17.54)	2.76 (1.02–7.46)*
Marital status	Never married	24	9	1	1
	Married	184	118	1.71 (0.76–3.80)	0.72 (0.24–2.11)
Residence	Urban	174	85	1	1
	Rural	34	42	2.52 (1.50–4.25)	1.68 (0.68–4.11)
Education	No read and write	31	47	1	1
	Grade 1–8	53	31	0.38 (0.20–0.72)	0.63 (0.27–1.46)
	Grade 9–10	59	23	0.25 (0.133–0.45)	0.46 (0.17–1.20)
	Certificate and above	65	26	0.26 (0.14–0.50)	0.54 (0.20–1.46)
Occupation	Employed	49	24	1	1
	Private worker	83	34	0.83 (0.44–1.57)	0.53 (0.23–1.24)
	Peasants	18	19	2.15 (0.96–4.83)	0.28 (0.07–1.09)
	House wife	34	31	1.86 (0.93–3.70)	1.15 (0.44–2.99)
	Other	24	19	1.61 (0.74–3.50)	0.37 (0.12–1.101)
Duration of T2DM	≤ 5 years	160	45	1	1
	> 5 years	48	82	6.07 (3.73–9.87)	4.09 (2.40–6.96)**
Medication	Drugs (OHA)	158	65	1	1
Regimen	Mixed(oral & insulin)	40	44	2.67 (1.59–4.48)	1.24 (0.56–2.390
	Insulin only	10	18	4.37 (1.91–9.98)	2.35 (0.82–6.66)
Hypertension	No	150	81	1	1
	Yes	58	46	4.93 (3.06–7.95)	3.52 (2.09–5.95)**
Physical activity	Good	87	40	1	1
	Poor	121	87	1.56 (0.98–2.48)	0.93 (0.50–1.72)
Adherence	No	46	20	1	1
	Yes	162	107	1.51 (0.85–2.71)	1.17 (0.55–2.47)

*OHA* oral hypoglycemic agent, *T2DM* type 2 diabetes mellitus, *OR* odds ratio, *COR* crude odds ratios, *AOR* adjusted odds ratios

Level of significance for factors * p-value < 0.05 and ** p-value < 0.001, Hosmer–Lemeshow goodness-of-fit (p = 0.424), (Variance inflation factor or VIF < 5). Variables such as marital status, residence, educational level, occupation, medication regimen, physical activity and adherence to diet had not significant association (p > 0.05)

## Discussion

Nowadays, diabetes mellitus is alarmingly increasing chronic disease that has short and long term sequels if early medication and life-time modification is not sought. Likewise, the disorder is growing faster and becoming serious medical problem in Ethiopia. Microvascular complications could lead to visual, renal and neurological malfunction that all together results in sever morbidity, mortality and negative socio-economic consequences. Hence, identifying associated factors of microvascular complications is very crucial to halt its irreversible consequences.

In the current study, the overall prevalence of microvascular diabetic complications was 37.9%. This proportion is in line with studies in Jimma, Ethiopia 41.5% [28], Metu, Ethiopia 38.5% [29], Ghana 35.3% [24], Saudi Arabia 35.4% [23], Brazil 41.6% [18]. However, this finding is higher than studies in Gondar Ethiopia 20.4% [25], Wollega, Ethiopia 31.2% [26] and Spain 25.2% [20]. On the other hand, this result is lower than studies in Debre Tabor, Ethiopia 43.9% [30], Gurage, Ethiopia 61% [27], Kuwait 61.6% [22], Middle east 45% [15], India 69% [31], China 57.5% [19], Bangladesh 50.4% [21], Greenland, 68% [12] and USA 77% [17]. The difference might be related to sample size, accessibility and advancement of health institutions, patient’s adherence to medication and practice to life style recommendations.

The current study concludes retinopathy (24.8%) is the commonest microvascular complication, and on the other hand, neuropathy (8.1%) is the least microvascular complication which is similar with findings from Debre Tabor, Ethiopia (26.4%, 8.3%) [30], Wollega, Ethiopia (20.7%, 9.8%) [26], Saudi Arabia (14.8%, 5.6%) [23], and Brazil (22.5%, 15.5%) [18] of retinopathy and neuropathy respectively.

In this study factors like increasing age of participants, duration of diabetes and co-morbid hypertension were significantly associated with the presence of microvascular complications.

The odds of developing at least one diabetes microvascular complications for age groups 60–87 yeas was 2.76 times higher as compared to younger diabetes aged 20–39 years. This result is supported by findings from Debre Tabor [30], Wollega [26], Gondar [25], Saudi Arabia [23], Kuwait [22], Bangladesh [21], and China [19]. This is probably due to with advanced age, there combined effect of insulin resistance and loss of beta cells that brings hyperglycemia which in turn causes microvascular damage. Individuals diagnosed with diabetes at advanced age, may not survive long enough for microvascular complications to develop and/or to become clinically noticeable [40]. Moreover, being older is a powerful predictor of vascular damages. They also develop premature complications and death as compared to younger age [41].

Participants who had a duration of diabetes more than 5 years was 4 times more likely to have microvascular complications in contrary to duration of diabetes 5 years or less. This result agreed with studies in Ethiopia (Wollega [26], Gurage [27], Mettu [29]), Ghana [24], Saudi Arabia [23], Kuwait [22], Greenland [12], Greece [9], Brazil [18], and USA [17]. Longer diabetic duration was a potential risk factor for both microvascular diseases and macro-vascular disease independently [16, 18, 42]. In parallel to diabetes longer duration, there is chronic asymptomatic hyperglycemia that ends up with susceptible organ damage at diagnosis [36].

Diabetes clients with co-morbid hypertension were 3.52 times more likely to acquire microvascular complications than non-hypertensive diabetes. This is similar with findings of Mettu, Ethiopia [29], Kuwait [22], Greece [9], China [19], Spain [18], Bangladesh [21], and USA [17]. The possible justification is that high blood pressure accelerates the progress and development of microvascular complications due to increased intracellular hyperglycemia through up regulation of the glucose transporter 1 [43] so that increased plasma glucose level leads to damage of retinal blood vessels and glomeruli, also impair regulation of retinal perfusion [17]. Indeed, hypertension had direct effect on retinal endothelial cell and function that causes cell growth and vasoconstriction which eventually predisposes patients to vascular complications [21, 44].

Unlike to this study, factors such as treatment regimen, poor physical activity and adherence to diet are significant for microvascular complication at Debre Tabor [30], Kuwait [22], Greenland [12], Bangladesh [21], and China [19].

As a limitation, the nature of cross-sectional study doesn’t show case-effect relationship. Moreover, we used document review that lacks completeness for patients’ profile. Also this study was an institution-based so that it is unable to generalize the findings to the entire populations. Recall bias is an expected additional limitation.

## Conclusions

The current study findings revealed that microvascular complications of diabetes were widespread particularly diabetic retinopathy that needs immediate intervention to halt its undesirable outcome of vision loss. Increasing age of participants, longer duration of diabetes and co-morbid hypertension were independent predictors of microvascular complications among type 2 diabetes mellitus. The study highlights the urgent need for strengthening and implementing of better diabetic management services at the area. Health professionals also should give more emphasis for diabetes mellitus through early screening and health education (primary prevention). Those aged diabetes patients with long duration of diabetes mellitus, and co-morbidities like hypertension should seek medication abruptly (secondary prevention). There is also a need to early detection and management of microvascular complication (tertiary prevention). Further better and high scale studies like prospective and clinical studies are highly recommended.

## Data Availability

In addition to the data and materials described in the manuscript, someone can get the dataset from the corresponding author MAS upon rational request.

## Abbreviations

AOR: Adjusted odd ratio
BMI: Body mass index
CI: Confidence level
COR: Crude odd ratio
DM: Diabetes mellitus
FBS: Fasting blood sugar
SPSS: Statistical Package for Social Sciences
